# Antisense Tissue Factor Oligodeoxynucleotides Protected Diethyl Nitrosamine/Carbon Tetrachloride-Induced Liver Fibrosis Through Toll Like Receptor4-Tissue Factor-Protease Activated Receptor1 Pathway

**DOI:** 10.3389/fphar.2021.676608

**Published:** 2021-05-11

**Authors:** Maha M. Shouman, Rania M. Abdelsalam, Mahmoud M. Tawfick, Sanaa A. Kenawy, Mona M. El-Naa

**Affiliations:** ^1^ Department of Pharmacology and Toxicology, Faculty of Pharmacy, Modern Sciences and Arts University (MSA), Giza, Egypt; ^2^ Department of Pharmacology and Toxicology, Faculty of Pharmacy, Cairo University, Cairo, Egypt; ^3^ Department of Biology, Faculty of Pharmacy, New Giza University, Giza, Egypt; ^4^ Department of Microbiology and Immunology, Faculty of Pharmacy (Boys), Al-Azhar University, Cairo, Egypt; ^5^ Department of Pharmacology and Toxicology, Faculty of Pharmacy, University of Sadat City, Sadat City, Egypt

**Keywords:** TF, PAR1, TLR4, liver fibrosis, inflammation, antisense oligodeoxynucleotides

## Abstract

Tissue factor (TF) is a blood coagulation factor that has several roles in many non-coagulant pathways involved in different pathological conditions such as angiogenesis, inflammation and fibrogenesis. Coagulation and inflammation are crosslinked with liver fibrosis where protease-activated receptor1 (PAR1) and toll-like receptor4 (TLR4) play a key role. Antisense oligodeoxynucleotides are strong modulators of gene expression. In the present study, antisense TF oligodeoxynucleotides (TFAS) was evaluated in treating liver fibrosis via suppression of TF gene expression. Liver fibrosis was induced in rats by a single administration of N-diethyl nitrosamine (DEN, 200 mg/kg; i. p.) followed by carbon tetrachloride (CCl4, 3 ml/kg; s. c.) once weekly for 6 weeks. Following fibrosis induction, liver TF expression was significantly upregulated along with liver enzymes activities and liver histopathological deterioration. Alpha smooth muscle actin (α-SMA) and transforming growth factor-1beta (TGF-1β) expression, tumor necrosis factor-alpha (TNF-α) and hydroxyproline content and collagen deposition were significantly elevated in the liver. Blocking of TF expression by TFAS injection (2.8 mg/kg; s. c.) once weekly for 6 weeks significantly restored liver enzymes activities and improved histopathological features along with decreasing the elevated α-SMA, TGF-1β, TNF-α, hydroxyproline and collagen. Moreover, TFAS decreased the expression of both PAR1 and TLR4 that were induced by liver fibrosis. In conclusion, we reported that blockage of TF expression by TFAS improved inflammatory and fibrotic changes associated with CCl4+DEN intoxication. In addition, we explored the potential crosslink between the TF, PAR1 and TLR4 in liver fibrogenesis. These findings offer a platform on which recovery from liver fibrosis could be mediated through targeting TF expression.

## Introduction

Liver fibrosis is a response to chronic liver injury induced by diverse causes such as infection, drugs, metabolic disorders, auto-immune diseases and cholestatic liver diseases (Borensztajn et al., 2010). Fibrogenesis is commonly associated with the generation of a chronic inflammation that results in an abnormal wound healing response. Hepatic stellate cells (HSCs) activation is the key pathogenic mechanism for the initiation and progression of liver fibrosis. A complex and tightly regulated cross-talks between HSCs, hepatocytes, Kupffer cells and sinusoidal endothelial cells (SECs) at the level of liver microcirculation are reported during fibrogenesis (Moreira, 2007).

Accumulation of collagen as well as other extracellular matrix (ECM) components in the liver leads to fibrous scar formation, which results in disruption of the liver architecture, hepatocyte loss, deterioration of the normal liver functions and ultimately liver failure (Karsdal et al., 2020). Liver fibrosis is a reversible process, as long as the liver is not at the stage of advanced cirrhosis (Benyon and Iredale, 2000). Given the highly dynamic process of liver fibrosis and the complex and multiple pathways involved in its progress, targeting one pathway that underlies the fibrotic process may not be sufficient to induce its reversal. However, through advances in the understanding of the key events and cellular pathways involved in the pathogenesis of fibrogenesis, the key targets for antifibrotic therapies are likely to be identified (Trautwein et al., 2015).

Recently, the association between chronic liver disease and coagulopathy is well established (Tripodi et al., 2011). Coagulation activity was recorded as a contributor to the pathogenesis of liver toxicity. Furthermore, it was reported that inflammation and coagulation are interrelated for fibrogenesis (Calvaruso et al., 2008).

Tissue factor (TF) is a 47-kDa transmembrane glycoprotein that is normally expressed throughout the body including the liver (Witkowski et al., 2016). TF is the main initiator of the protease coagulation cascade *in vivo*, leading to thrombin generation (Rauch and Nemerson, 2000). It has been reported that under pathological conditions TF expression can be upregulated by many inducers such as cytokines, hypoxia and mechanical injury, thus concentrating TF to the sites of injury (Giesen et al., 1999). TF mediates different pathological signal cascades via a family of G-protein coupled receptors called protease-activated receptors (PARs) (Rao and Pendurthi, 2005).

Four types of PARs have been identified PAR1, PAR2, PAR3 and PAR4. PAR1 receptor was found in the normal liver as well as diseased liver (Rullier et al., 2008; Nault et al., 2016) and has been shown to perform a key role in the pathogenesis of liver fibrosis (Sullivan et al., 2010). PAR1 is expressed by various cells including endothelial cells, fibroblasts, smooth muscle cells and T lymphocytes (Coughlin, 2000). In the liver of patients with cirrhosis and chronic hepatitis, PAR1 was positively stained in fibroblasts in the fibrotic septa in addition to the inflammatory cells infiltrated around newly formed blood vessels and bile ducts (Rullier et al., 2008). PAR1 is primarily activated by thrombin and can be also activated by activated protein C (Riewald et al., 2003), coagulation factor Xa (FXa; Riewald et al., 2001), coagulation factor VIIa (FVIIa; Camerer et al., 2000) and plasmin (Pendurthi et al., 2002). Thrombin-mediated activation of PAR1 has been reported to contribute to several inflammatory and fibrotic diseases including liver fibrosis (Sullivan et al., 2010).

In the liver, toll-like receptor 4 (TLR4) is found on both Kupffer cells and HSCs (Bai et al., 2013). TLR4 is the main target for lipopolysaccharide (LPS) and is largely involved in the inflammatory reaction associated with liver fibrosis (Beutler, 2004). TLR4 signaling through Kupffer cells leads to the release of proinflammatory cytokines such as TNF-α, IL-1 and IL-6 (Beutler, 2004). Moreover, TLR4 expressed on Kupffer cells is involved in the fibrogenic signaling of HSCs and enhancing their response to transforming growth factor-1β (TGF-1β) thus promoting liver fibrosis (Seki et al., 2007). Interestingly, it has been reported that in patients with hepatitis C infection, specific single-nucleotide polymorphisms of TLR4 affected the rate of fibrosis progression (Huang et al., 2007).

Blocking of specific gene expression has recently gained a growing considerable interest as a tool to decrease the expression of a target protein. DNA encodes RNA, which is then translated into proteins (Chery, 2016). Antisense oligodeoxynucleotides (AS-ODNs) are single-stranded oligodeoxynucleotides that bind to compatible mRNA with a high degree of accuracy, leading to a decline in the target protein level (Bennett and Swayze, 2010). Different types of oligonucleotides antisense sequences, including reverse sequences, antisense sequences containing one or more mismatches and scrambled oligonucleotides have been used as a control of AS-ODNs (Gagnon and Corey, 2019). Over the last years, therapeutic strategies including AS-ODNs that suppress translation of mRNA and other oligonucleotides that interfere with RNA pathway have been substantially improved as a therapy platform at both preclinical and clinical levels (Chi et al., 2005; Kastelein et al., 2006). In 1998, fomivirsen was approved as the first agent that inhibits the translation of mRNA encoding for cytomegalovirus at its early immediate region proteins and permitted for treating cytomegalovirus retinitis (Jabs and Griffiths, 2002). Importantly, by January 2020, 10 oligonucleotide drugs have gained regulatory approval from the FDA (Roberts et al., 2020).

The objective of this work is to study the theory that chemically induced liver fibrosis is TF-dependent and consequently inhibition of TF expression by antisense tissue factor oligodeoxynucleotides (TFAS) could be associated with reduced severity of liver fibrosis. In addition, the study aimed at exploring the TLR4-TF-PAR1 signaling loop as a novel pathway that may be involved in liver fibrosis and the possible role of suppressing TF expression in blocking this loop as a form of cross-talk between coagulation, inflammation and fibrogenesis.

## Material and Methods

### Experimental Animals

Male Sprague Dawley rats with an average weight of 120–150 gm (5–6 weeks old) were purchased from the Egyptian Organization for Biological Products and Vaccines (Cairo, Egypt). Animals were placed in cages and kept under conventional laboratory conditions throughout the study (room temperature 24–27°C and 55 ± 10% humidity) with alternating 12 h light and dark cycles. Animals were fed normal chow and were permitted water *ad libitum*. They were left in the animal house at Faculty of Pharmacy, Cairo University for acclimatization for one week before the start of the study where rats whose weight exceeded 150 gm were excluded. Male rats are strictly used while female rats are avoided in the experiment to prevent unintended risks. The experimental protocol was approved by the Research Ethics Committee (REC) of Faculty of Pharmacy, Cairo University (PT 1902).

### Drugs and Chemicals

N-diethyl nitrosamine (DEN) was purchased from Sigma Chemicals (MO, United States) and dissolved in saline. Carbon tetrachloride (CCl4) was obtained from El Gomhorya Co. (Cairo, Egypt). Tissue factor oligodeoxynucleotides (TF-ODNs) were purchased from Integrated DNA Technologies (San Diego, CA, United States) and dissolved in saline. The sequence of the rat antisense tissue factor oligodeoxynucleotides (TFAS) is 5’-CAT​GGG​GAT​AGC​CAT-3’ while the sequence of scrambled control of tissue factor oligodeoxynucleotides (TFSC) is 5’-TGA​CGC​AGA​GTC​GTA-3’. All chemicals were of the highest purity and analytical grade.

### Sample Size Calculation

A total of 40 rats were divided into five groups (*n = 8*) where each group was placed in a cage. The sample size was calculated using G*Power software (GPower 3.1. Ink) where the effect size is 0.77, α level is 0.05 and power (1-β) is 0.95.

### Experimental Design

Rats were allocated to cages by simple randomization using a web-based research randomizer and within the same cage, rats received the treatment randomly. The technician was blinded to the group status and the treatment administered to the rats. All the treatments and animals were handled and monitored in the same way. The authors were responsible for treatment preparation, anesthesia and sample collection. The groups were divided as follows:

Control group: healthy rats received saline throughout the experiment, TFAS group: rats treated with TFAS (2.8 mg/kg; s. c.) once a week for six weeks according to Nakamura et al. (2002) and Shimizu et al., (2014).

DEN+CCl4 group: liver fibrosis was induced by injection of a single dose of DEN (200 mg/kg; i. p.) and after one week CCl4 was administered (3 ml/kg; s. c.) once a week for six weeks according to Mansour et al. (2010) with some modification.

TFSC+DEN+CCl4 group: rats intoxicated with DEN and received TFSC (2.8 mg/kg; s. c.) concomitantly with CCl4 for 6 weeks.

TFAS+DEN+CCl4 group: rats intoxicated with DEN and received TFAS (2.8 mg/kg; s. c.) concomitantly with CCl4 for 6 weeks.

Animals were sacrificed following the time frame from starting the experiment till the end of the 7th week.

### Sample Collection

Blood samples were collected from the jugular vein and sera were separated. Rats were euthanized using thiopental (85 mg/kg; i. p.; Gonca, 2015). The liver was divided into 2 parts; one part was kept in 10% neutral formalin, whereas the second part was stored in −80°C. The measurements were performed by personnel that were blinded completely to the group status.

### Assessment of Liver Functions

Serum liver enzymes activities; alanine aminotransferase (ALT), aspartate aminotransferase (AST) and alkaline phosphatase (ALP) were assessed using colorimetric commercially available assay kits (Biodiagnostic, Giza, Egypt) according to the manufacturer's instructions.

### Determination of Liver Content of Tumor Necrosis Factor-alpha

Part of the frozen liver tissue was homogenized in phosphate buffer saline (PBS, pH 7.4) for the assessment of liver TNF-α content using ELISA specific kit (Ray Biotech, United States; ELR-TNFα). The parameter was assessed according to the manufacturer's protocol.

### Determination of Liver Hydroxyproline Content

Liver hydroxyproline content was assessed using frozen liver tissue as previously described by Edwards and O’Brien (1980) with slight modifications. Briefly, liver samples were weighed and hydrolyzed in 2.5 ml of 6N HCl at 110°C for 18 h in Teflon coated tubes. The hydrolysate was centrifuged at 3000 rpm for 10 min; the pH of the supernatant was allocated to 7.4 and the absorbance was measured at 558 nm. Total hydroxyproline content was measured against a standard curve established with trans-4-hydroxy-l-proline (Sigma- Aldrich, St Louis, MO, United States).

### Analysis of Tissue Factor, Transforming Growth Factor-1beta and Protease-Activated Receptors1 Gene Expression *via* qRT-PCR

Relative quantification of gene expression was performed by extraction of RNA from liver cells using TRIzol plus RNA purification kit (life technologies, Carlsbad, United States) according to the manufacturer’s protocol. RNA was reverse transcribed using High Capacity cDNA Reverse Transcription Kit (Applied Biosystems, Foster, CA, United States). Quantification of TGF-1β and PAR1 PCR was carried out using Rotor-Gene Q 5 plex real-time Rotary analyzer (Corbett Life Science, United States). Quantification of TF RNA, TGF-1β RNA and PAR1 RNA was carried out using PCR fluorescence quantitative diagnostic kit, with SYBR Green PCR Master Mix (Applied Biosystems, United States). For quantification of TGF-1 β, the primers 5’-TAC​CAT​GCC​AAC​TTC​TGT​CTG​GGA-3’ (forward primer) and 5’-ATG​TTG​ACA​ACT​GCT​CCA​CCT​TG-3’ (reverse primer) were normalized against β-actin 5’-ATC​TGG​CAC​CAC​ACC​TTC-3’ (forward primer) and 5’-AGC​CAG​GTC​CAG​ACG​CA-3’ (reverse primer). For quantification of PAR1, primers 5’-CTT​GCT​GAT​CGT​CGC​CC-3’ (forward primer) and 5’-TTC​ACC​GTA​GCA​TCT​GTC​CT-3’ (reverse primer) and for quantification of TF, primers 5’-ATG​GCT​ATC​CCC​ATG-3’ (forward primer) and 5’-CAT​GGG​GAT​AGC​CAT-3’ (reverse primer) were normalized against GADPH 5’-CTG​CAC​CAC​CAA​CTG​CTT​AC-3’ (forward primer) and reverse 5’-CAG​AGG​TGC​CAT​CCA​GAG​TT-3’ (reverse primer).

### Flow Cytometric Analysis of Toll-Like Receptor4

For detection of TLR4 cell surface expression, frozen liver tissue was homogenized then single-cell suspension was washed with staining buffer (PBS containing 1% FBS). Cells were then incubated with biotin-conjugated rat anti-human TLR4 antibody at a concentration of 20 ml/10^6^ cells for 30 min on ice. After washing with staining buffer, the cells were mixed with Streptavidin-phycoerythrin and immediately analyzed with a flow cytometer FACScan and CellQuest Software.

### Histopathological Examination of Liver With Collagen and Fibrosis Scoring

Liver samples preserved in 10% neutral formalin were washed under tap water; then serial dilutions of alcohol were used for dehydration. Specimens were cleared in xylene and embedded in paraffin. Sections at 4 μm thicknesses were prepared by slide microtome and stained with hematoxylin and eosin (H and E) and Masson’s Trichome staining to examine liver histopathological and fibrotic changes. All histopathological examinations were performed by an experienced pathologist who was blinded to the study groups. All methods of tissue sample preparation and staining were carried out as outlined by Drury and Wallington (1983). Qualitative and quantitative scoring of collagen was performed using a full HD microscopic imaging system (Leica Microsystems GmbH, Germany) operated by Leica Application software. The total specimen area and the blue-stained pixels (representing collagen) were segmented. Percent (%) collagen was calculated as the ratio of blue-stained to total specimen pixels. The criteria used for microscopic lesions and fibrosis scoring was listed as follows (Al-Sayed et al., 2019): (−), no lesions were demonstrated; (+), few lesions were demonstrated in one examines section; (++), mild lesions were focally demonstrated in some examined sections; (+++), moderate lesions were diffusely demonstrated in some examined sections; and (++++), severe lesions were diffused in all examined sections.

### Immunohistochemical Staining of Alpha Smooth Muscle Actin and Tissue Factor in the Liver

According to the manufacturer’s protocol, deparaffinized 4 μm thick tissue sections were treated with 3% H_2_O_2_ for 20 min, washed, then incubated with anti-alpha smooth muscle actin antibody and anti-tissue factor antibody overnight. Tissue sections were washed with PBS followed by incubation with secondary antibody HRP Envision kit (DAKO) for 20 min and incubated after washing with diaminobenzidine (DAB) for 10 min and then washed finally with PBS and hematoxylin was added for counter staining. Finally, tissue sections were dehydrated and cleared in xylene. Area percentage of immune-expression levels of α-SMA and TF sections were determined using Leica application module for tissue sections analysis attached to full HD microscopic imaging system (Leica Microsystems GmbH, Germany).

### Statistical Analysis

Data analysis was carried out with complete blindness to the study group status. Results were expressed as mean ± SEM. Statistical significance was determined by one-way ANOVA followed by Tukey-Kramer post-Hoc test. P value < 0.05 was considered significant. In addition, correlation and linear regression between TF and the assessed parameters as well as between PAR1 and TLR4 were carried out where slope differences were compared, tested and checked for significance at P< 0.0001. Correlation Coefficient “r” was calculated where the difference in “r” value states that variation of one of the variables will affect the variation in the other one through *R* ^2^ calculation. GraphPad Prism 8 for Windows (GraphPad Software, Inc, La Jolla, United States) was used in all analyses.

## Results

### Effect of Tissue Factor-Oligodeoxynucleotides on the Liver Tissue Factor Expression

**FIGURE 1:**
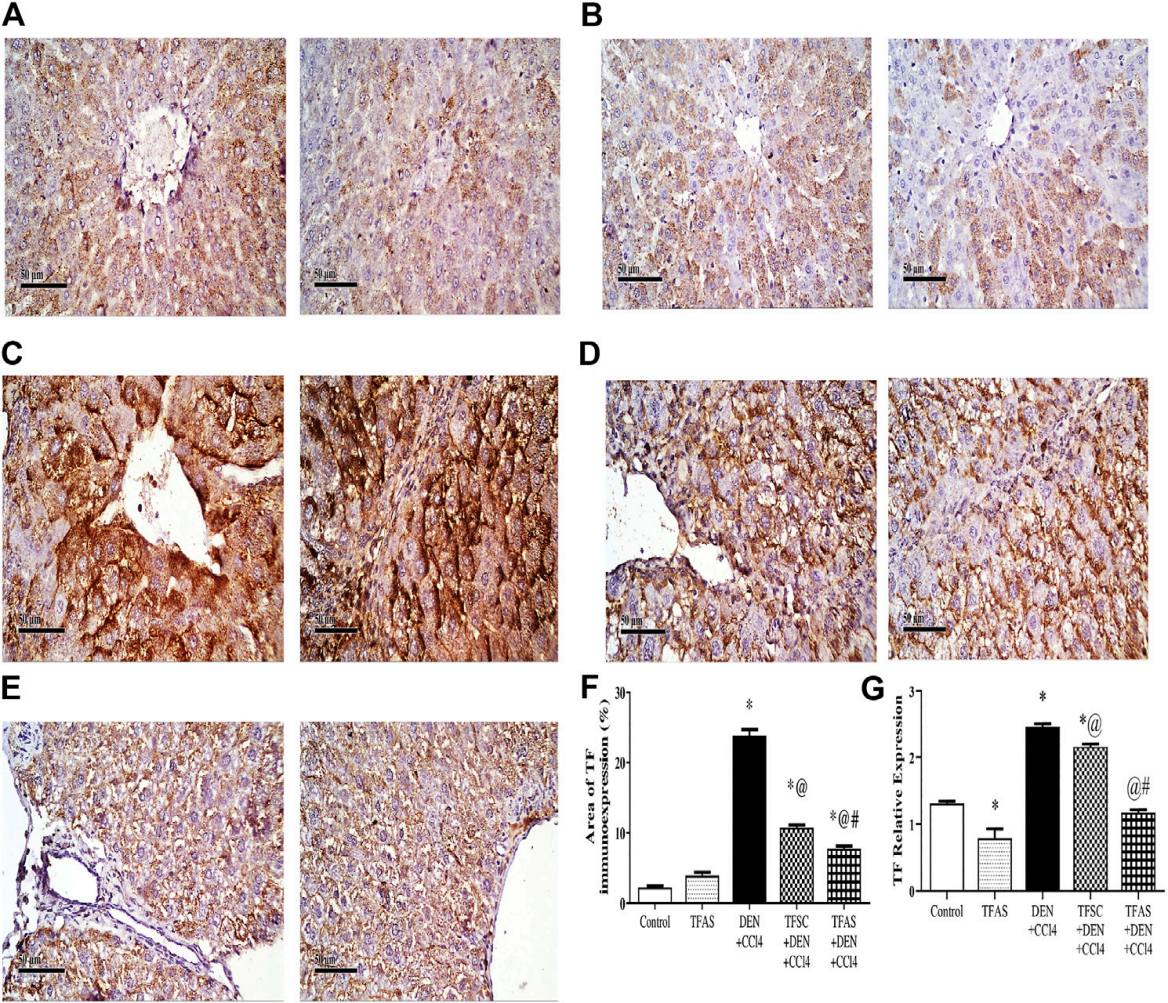
Effect of TF-ODNs on the liver TF expression in DEN+CCl4 intoxicated rats. Immune-stained liver section of TF expression with positive stained grades of cytoplasmic and sub membranous positivity in numerous hepatocytes, **(A)** control group, **(B)** TFAS group, **(C)** DEN+CCl4 group, **(D)** TFSC+DEN+CCl4 group, **(E)** TFAS+DEN+CCl4 group and **(F)** The percentage area of TF immune-expression and **(G)** qPCR determined TF expression in all study groups. Data are expressed as mean ± SEM (n = 8). (*), (@) and (#) indicate significant difference from Control, DEN+CCl4 and TFSC+DEN+CCl4, respectively at *P* < 0.05 using one-way ANOVA followed by Tukey-Kramer post-Hoc test. TF: tissue factor; DEN: N-diethyl nitrosamine; CCl4: carbon tetrachloride; TFSC: scrambled tissue factor oligodeoxynucleotides; TFAS: antisense tissue factor oligodeoxynucleotides.

Immunohistochemical detection of TF expression in liver sections of control and TFAS groups showed weak basal expression of TF (Figures 1A,B). However, liver sections of DEN+CCl4 intoxicated rats showed sharply stained positive grades of cytoplasmic and sub membranous positivity in numerous hepatocytes (Figure 1C). The liver sections from DEN+CCl4 intoxicated rats received TFSC showed decreased hepatocellular staining compared to the DEN+CCl4 intoxicated group (Figure 1D). Rats that received TFAS showed a profound decrease in TF expression (Figure 1E). Area of TF expression showed a significant increase in the DEN+CCl4 intoxicated rats by 943.48% comparing to the basal expression of the control group. Treatment of rats with TFSC and TFAS resulted in a significant decrease in the elevated TF expression by 54.16 and 67.92%, respectively in comparison to untreated intoxicated rats. TFAS treatment significantly decreased TF expression by 30% compared to TFSC treatment (Figure 1F).

TF expression was determined by qPCR that showed a significant decrease by 39.78% upon treatment with TFAS alone when compared to the control group. TF expression increased significantly in the DEN+CCl4 intoxicated group by 88.03% compared with control group. Treatment of rats with TFSC and TFAS decreased the elevated TF expression by 12.17 and 52.37%, respectively compared with intoxicated group. Intoxicated rats that were treated with TFAS treatment showed a significant decrease by 45.78% when compared to TFSC treatment (Figure 1G).

### Effect of Tissue Factor-Oligodeoxynucleotides on Protease-Activated Receptors1 Expression in Liver

**FIGURE 2:**
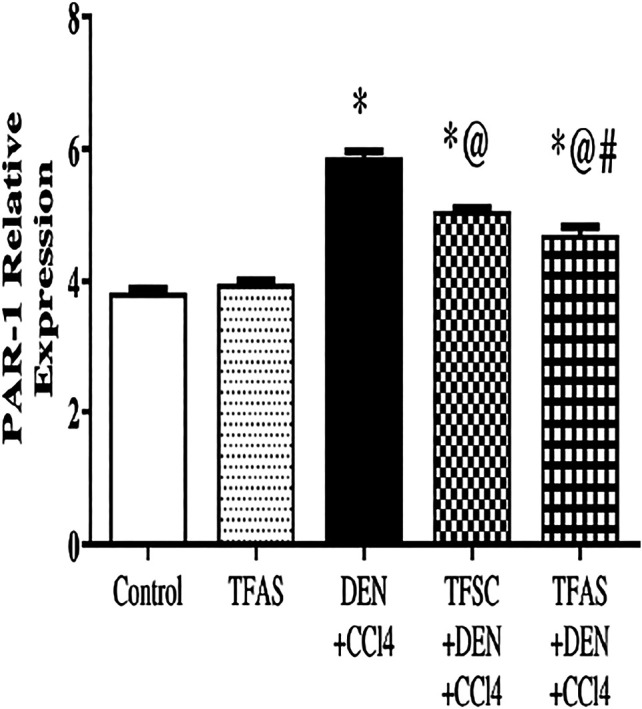
Effect of TF-ODNs on relative expression of PAR1 in liver. Data are expressed as mean ± SEM (n = 8). (*), (@) and (#) indicate significant difference from Control, DEN+CCl4 and TFSC+DEN+CCl4, respectively at *P* < 0.05 using one-way ANOVA followed by Tukey-Kramer post-Hoc test. PAR1: protease activated receptor 1; DEN: N-diethyl nitrosamine; CCl4: carbon tetrachloride; TFSC: scrambled tissue factor oligodeoxynucleotides; TFAS: antisense tissue factor oligodeoxynucleotides.

PAR1 expression in the livers excised from DEN+CCl4 intoxicated rats was significantly increased by 55.26% compared to the control group. The treatment of intoxicated rats with TFSC and TFAS significantly decreased the elevated level of PAR1 by 13.56 and 20.34%, respectively compared to the DEN+CCl4 group and TFAS treatment manifested a significant decrease in PAR1 expression by 7.84% compared to TFSC (Figure 2).

### Effect of Tissue Factor-Oligodeoxynucleotides on Liver Alpha-Smooth Muscle Actin Expression

**FIGURE 3:**
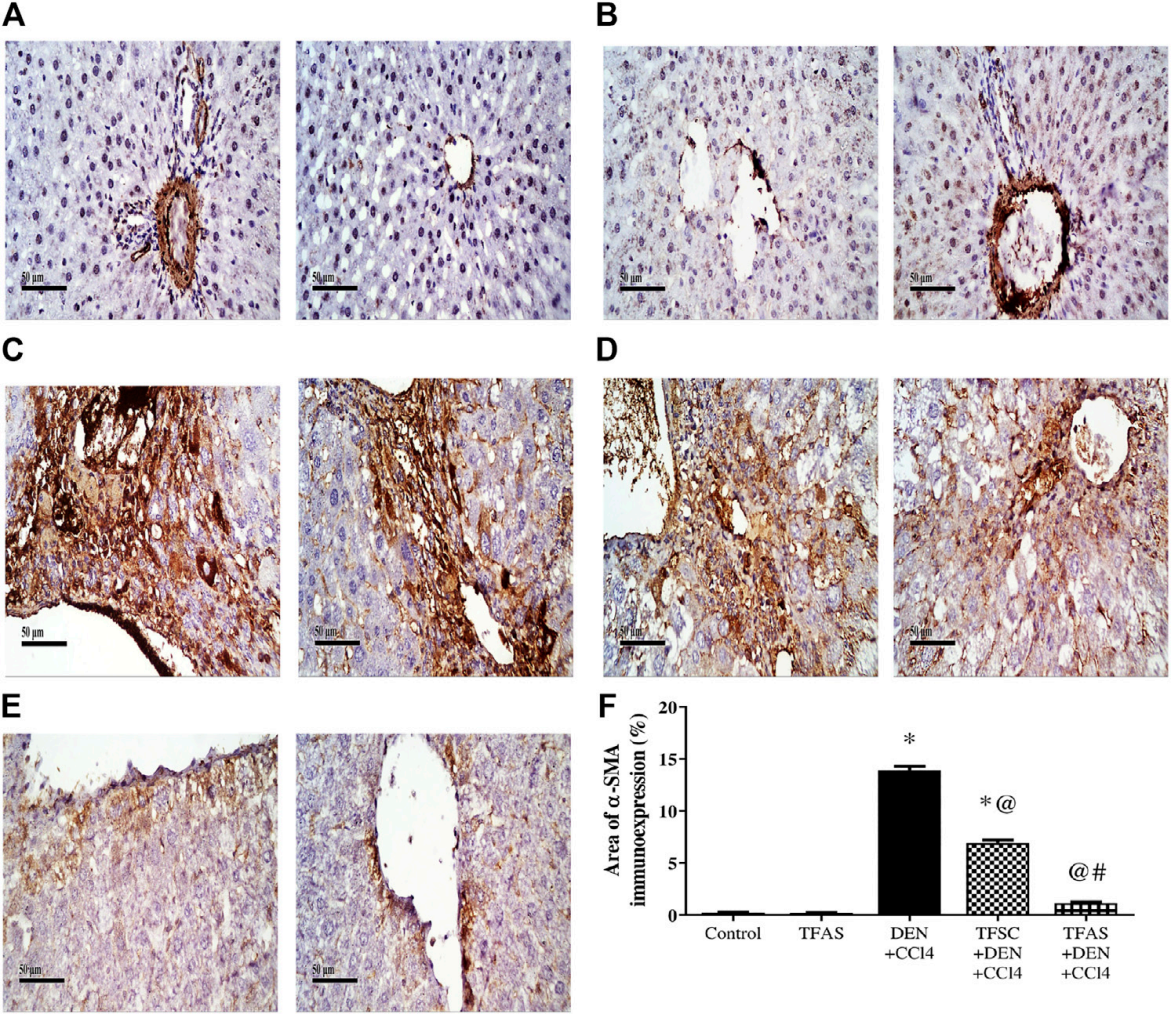
Effect of TF-ODNs on liver α-SMA expression. Immune-stained liver section of α-SMA expression is detected in hepatic stellate cells, fibroblasts and vascular wall, **(A)** control group, **(B)** TFAS group, **(C)** DEN+CCl4 group, **(D)** TFSC+DEN+CCl4 group, **(E)** TFAS+DEN+CCl4 group and **(F)** The percentage of α-SMA expression. Data are expressed as mean ± SEM (n = 8). (*), (@) and (#) indicate significant difference from Control, DEN+CCl4 and TFSC+DEN+CCl4, respectively at *P* < 0.05 using one-way ANOVA followed by Tukey-Kramer post-Hoc test. α-SMA: alpha smooth muscle actin; DEN: N-diethyl nitrosamine; CCl4: carbon tetrachloride; TFSC: scrambled tissue factor oligodeoxynucleotides; TFAS: antisense tissue factor oligodeoxynucleotides.

Control and TFAS treated animals showed weak basal expression of α-SMA (Figures 3A,B). Rats intoxicated with DEN+CCl4 showed increased α-SMA expression with strongly stained hepatic cells, fibroblasts and vascular wall (Figure 3C). DEN+CCl4 intoxicated group treated with TFSC revealed a decreased area of α-SMA positive cells (Figure 3D). In the DEN+CCl4 intoxicated group treated with TFAS, the area of α-SMA expression was nearly similar to that of control animals (Figure 3E). Area of α-SMA stained liver cells showed a significant decrease with both TFSC and TFAS treatment when compared to DEN+CCl4 intoxicated animals by 50.71 and 91.43%, respectively. In addition, TFAS treatment showed a significant decrease in α-SMA expression by 82.61% compared to TFSC treatment of intoxicated rats (Figure 3F).

### Effect of Tissue Factor-Oligodeoxynucleotides on Serum Activities of Alanine aminotransferase, Aspartate aminotransferase and Alkaline Phosphatase

**FIGURE 4:**
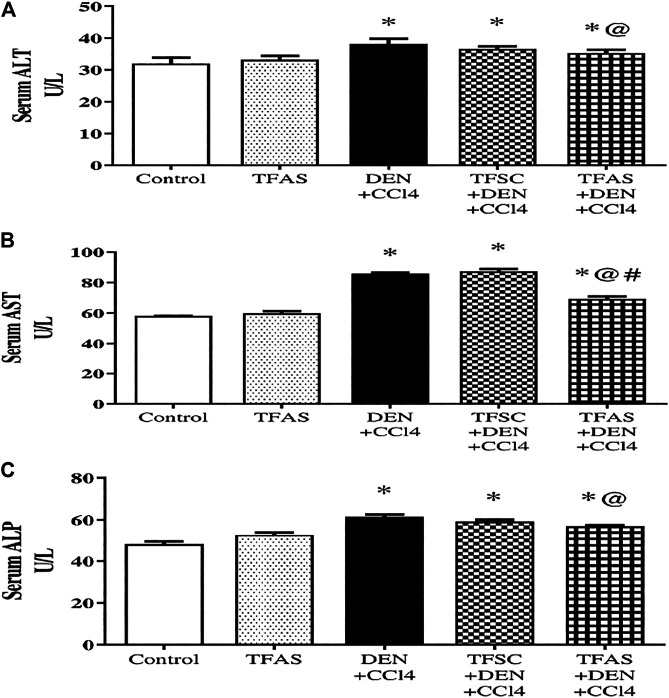
Effect of TF-ODNs on serum activities of ALT, AST and ALP enzymes. Liver enzymes activities of ALT, AST and ALP are presented in figure **(A)**, **(B)** and **(C)**, respectively. Data are expressed as mean ± SEM (n = 8). (*), (@) and (#) indicate significant difference from Control, DEN+CCl4 and TFSC+DEN+CCl4, respectively at *P* < 0.05 using one-way ANOVA followed by Tukey-Kramer post-Hoc test. ALT: alanine aminotransferase; AST: aspartate aminotransferase; ALP: alkaline phosphatase; DEN: N-diethyl nitrosamine; CCl4: carbon tetrachloride; TFSC: scrambled tissue factor oligodeoxynucleotides; TFAS: antisense tissue factor oligodeoxynucleotides.

As shown in Figure 4, serum activities of ALT **(A)**, AST **(B)** and ALP **(C)** are elevated significantly upon DEN+CCl4 intoxication by 18.75, 48.28 and 29.17%, respectively compared to the control group. These elevations were significantly decreased in rats intoxicated with the DEN+CCl4 and treated with TFAS by 7.89, 19.77 and 8.06%, respectively, compared to the untreated intoxicated rats. There was a non-significant difference in serum enzymes activities between the DEN+CCl4 group and that treated with TFSC. On the other hand, serum activity of AST was significantly improved in the intoxicated animals treated TFAS compared to those treated with TFSC.

### Effect of Tissue Factor-Oligodeoxynucleotides on Histopathological Features

**FIGURE 5:**
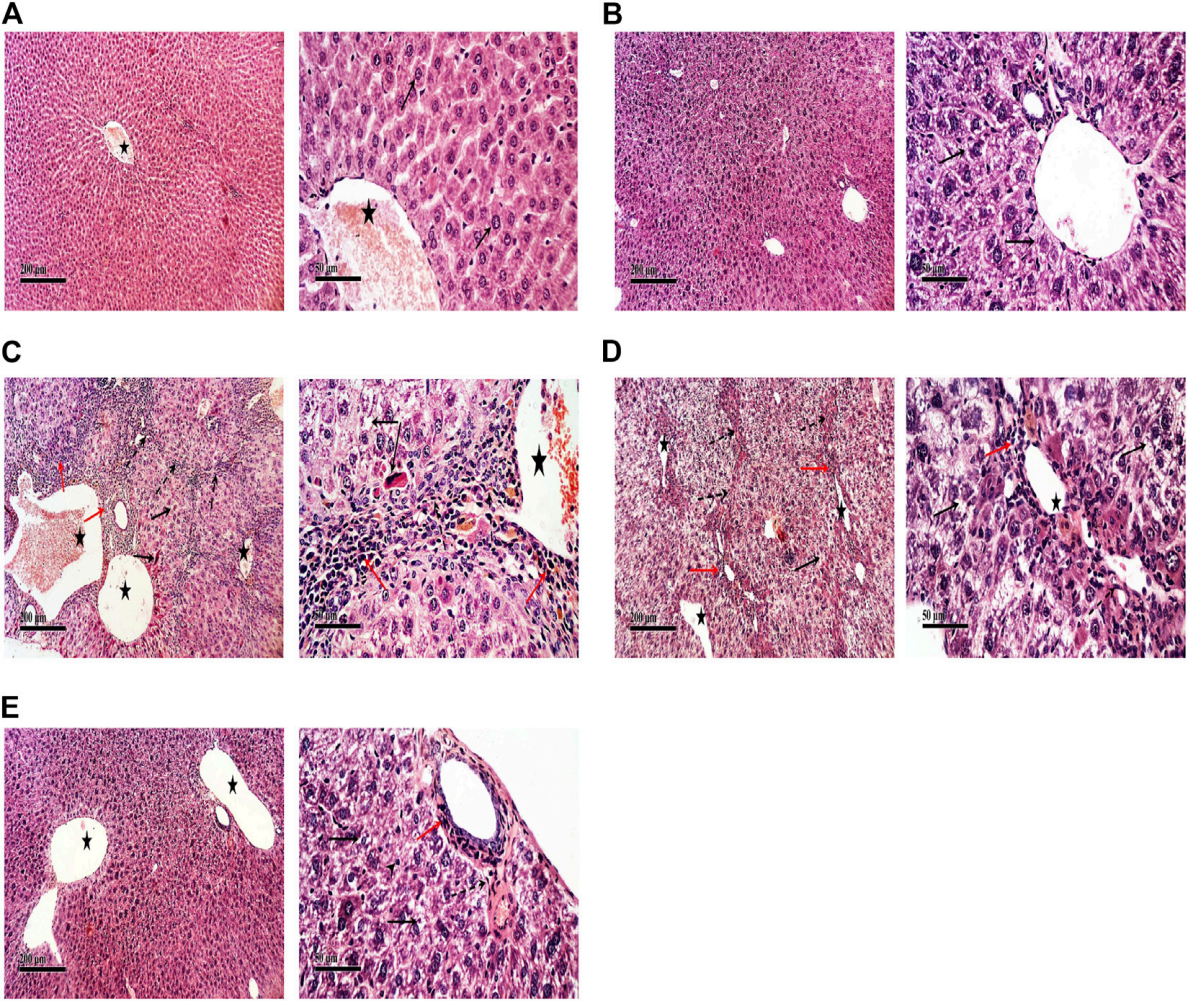
Effect of DEN+CCl4 and TF-ODNs on histopathological features. **(A)** normal histologic structure showing central vein (star) from control group, **(B)** TFAS treated rats showing apparently normal histological features with minimal degenerative changes, **(C)** DEN+CCl4 intoxicated rats showing sever vacuolar degenerative and necrotic hepatocellular changes (black arrow) accompanied with many dilated liver blood vessels (star) and sever periportal inflammatory cells infiltrates (red arrow), **(D)** TFSC treated intoxicated rats showing slight amelioration of abnormal morphological changes with persistence of sever vacuolar degenerative of hepatocytes (arrow), **(E)** TFAS treated intoxicated rats showing moderate amelioration of morphological changes with dilated blood vessels and mild periportal inflammatory cells infiltrates (arrow head); DEN: N-diethyl nitrosamine; CCl4: carbon tetrachloride; TFSC: scrambled tissue factor oligodeoxynucleotides; TFAS: antisense tissue factor oligodeoxynucleotides.

The control group of rats showed normal histology of the liver (Figure 5A) as well as apparent normal features in TFAS-treated rats (Figure 5B). However, rats intoxicated with DEN+CCl4 showed a severe loss in hepatic architecture (Figure 5C) where degeneration of hepatocytes, area of coagulative hepatocellular necrosis, sinusoidal dilatation, the proliferation of biliary epithelium with periportal infiltration of inflammatory cells in portal areas as well as fibroblastic proliferation and bridging were demonstrated in this group (Table 1). These histopathological alterations were moderately reduced in the group intoxicated and treated with TFAS (Figure 5E). In contrast, treatment with TFSC didn’t significantly improve the histopathological features (Figure 5D).

**TABLE 1:** Effect of TF-ODNs on liver histopathological and fibrotic changes.

Groups	Degenerative/necrotic changes	Inflammatory cells infiltrates	Fibroblastic proliferation and bridging	Dilated blood vessels
Control	−	−	−	−
TFAS	+	−	−	−
DEN+CCl4	++++	++++	++++	+++
TFSC+DEN+CCl4	++++	+++	+++	+++
TFAS+DEN+CCl4	+++	++	+	+++

− nil: no lesions were demonstrated; +: few lesions were demonstrated in one examined section; ++: mild lesions were focally demonstrated in some examined section; +++: moderate lesions were diffusely demonstrated in some examined section; ++++: severe lesions were diffused in all examined sections.

### Effect of Tissue Factor-Oligodeoxynucleotides on Collagen Deposition and Liver Hydroxyproline Content

**FIGURE 6:**
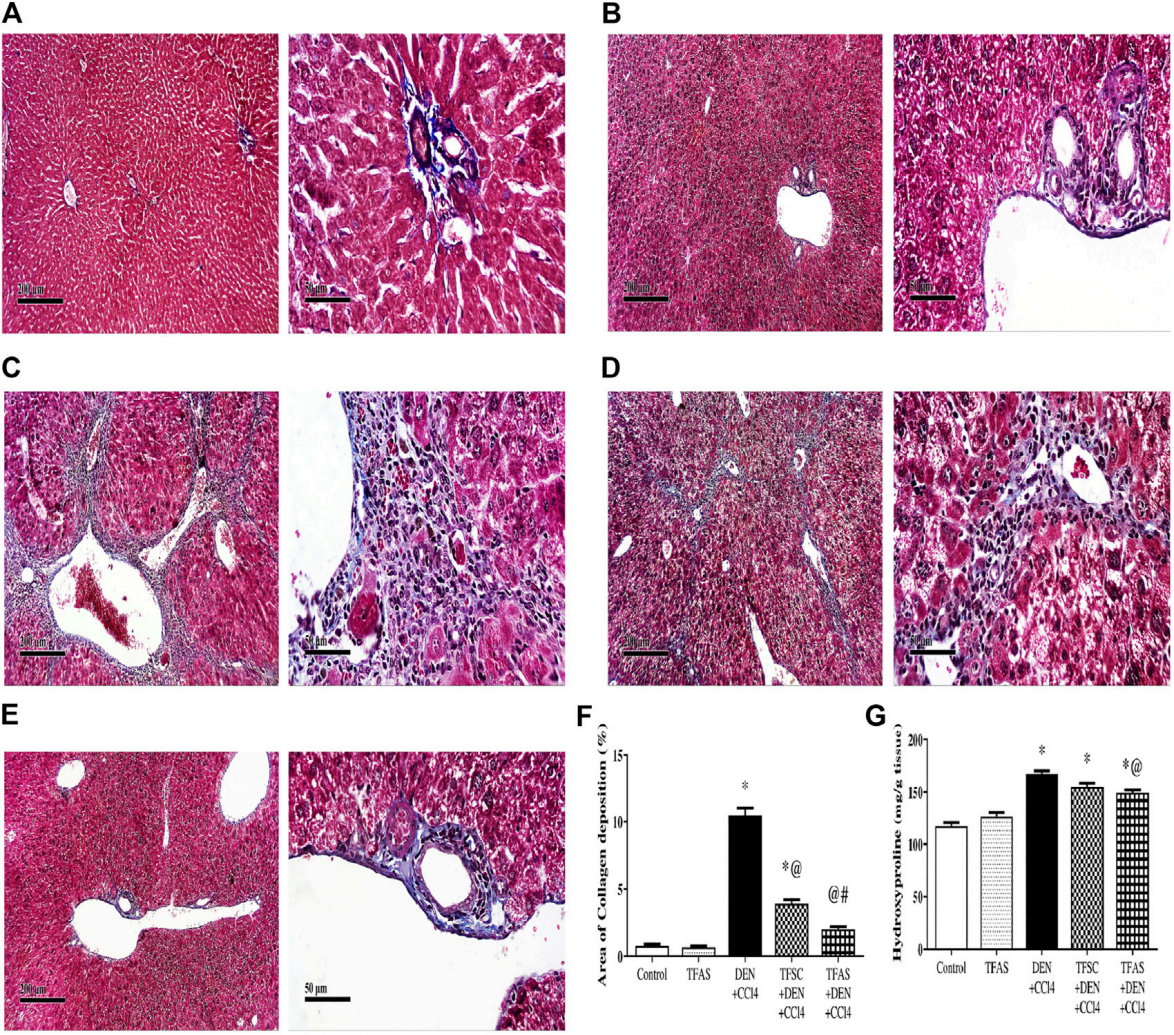
Effect of TF-ODNs on collagen deposition and hydroxyproline content in the liver. **(A)** Masson’s Trichome-stained liver section of control group, **(B)** TFAS group, **(C)** DEN+CCl4 group where obvious periportal proliferation of fibroblasts are detected as well as bridging of fibroblasts with abundant formation of collagen fibers, **(D)** TFSC+DEN+CCl4 group and **(E)** TFAS+DEN+CCl4 group showed significant reduction of activated fibroblasts and collagen fibers, **(F)** Percentage area of collagen deposition and **(G)** liver hydroxyproline content in different study groups. Data are expressed as mean ± SEM (n = 8). (*), (@) and (#) indicate significant difference from Control, DEN+CCl4 and TFSC+DEN+CCl4, respectively at *P* < 0.05 using one-way ANOVA followed by Tukey-Kramer post-Hoc test. DEN: N-diethyl nitrosamine; CCl4: carbon tetrachloride; TFSC: scrambled tissue factor oligodeoxynucleotides; TFAS: antisense tissue factor oligodeoxynucleotides.

Control and TFAS-treated normal rats showed uniform collagen distribution (Figures 6A,B). Animals intoxicated with DEN+CCl4 showed central vein, portal tract and septal fibrosis that were stained positively for collagen fiber bundles in Masson’s Trichome stained liver sections (Figure 6C). Treatment with TFSC showed relatively decreased area of collagen accumulation in liver sections (Figure 6D) but animals treated with TFAS after intoxication restored collagen distribution nearly to the control rats (Figure 6E). The elevated area of collagen deposition in rats intoxicated with DEN+CCl4 was significantly declined with treatment with TFSC and TFAS by 61 and 80%, respectively. In addition, TFAS showed a more significant decrease by 48.72% compared to rats treated with TFSC (Figure 6F). Animals intoxicated with DEN+CCl4 showed a significant increase in liver hydroxyproline content by 42.7% compared to the control group. In contrast to collagen deposition, treatment with TFSC didn’t show significant difference in hydroxyproline content, while TFAS showed a significant decrease by 10.68% compared to DEN+CCl4 intoxicated group (Figure 6G).

### Effect of Tissue Factor-Oligodeoxynucleotides Effect on Toll-Like Receptor4 Expression in the Liver

**FIGURE 7:**
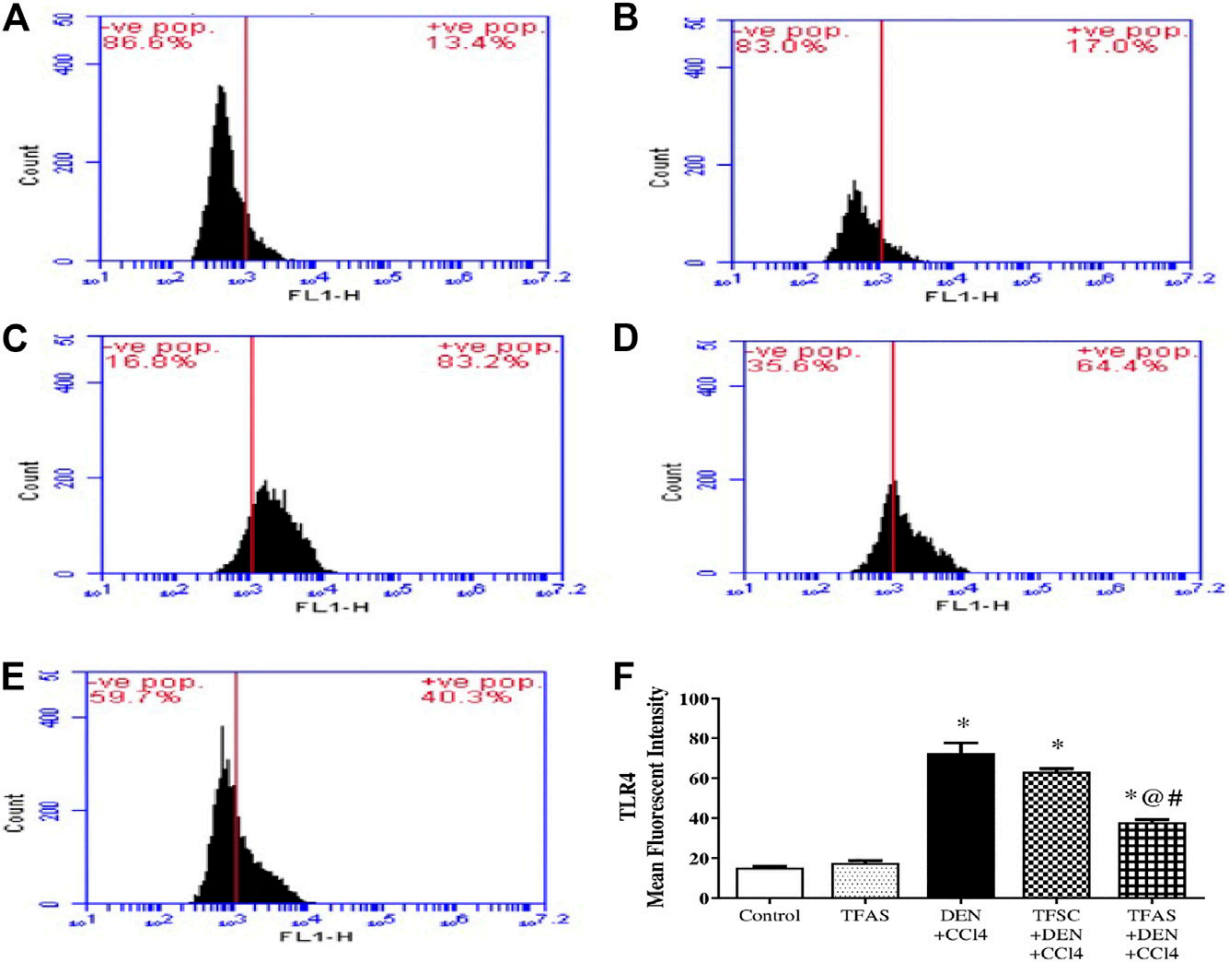
Flow cytometric analysis of the effect of TF-ODNs on TLR4 expression in the liver. Histogram plots of **(A)** Control group, **(B)** TFAS group, **(C)** DEN+CCl4 group, **(D)** TFSC+DEN+CCl4, **(E)** TFAS+DEN+CCl4 and **(F)** Mean fluorescence intensity of TRL4. Data are expressed as mean ± SEM (n = 8). (*), (@) and (#) indicate significant difference from Control, DEN+CCl4 and TFSC+DEN+CCl4, respectively at *P* < 0.05 using one-way ANOVA followed by Tukey-Kramer post-Hoc test. TLR4: toll like receptor 4; DEN: N-diethyl nitrosamine; CCl4: carbon tetrachloride; TFSC: scrambled tissue factor oligodeoxynucleotides; TFAS: antisense tissue factor oligodeoxynucleotides.

Flow cytometric analysis revealed low TLR4 expression on liver cells in the control group as well as TFAS treated normal rats (Figures 7A,B,F). Rats intoxicated with DEN+CCl4 showed significant increase in TLR4 expression by 386.67% (Figures 7C,F). The intoxicated group treated with TFAS showed a significant reduction in the expression of TLR4 by 47.95% (Figures 7E,F). Also, TLR4 expression was significantly decreased in intoxicated group with TFAS treatment by 39.68% compared to TFSC treated one.

### Effect of Tissue Factor-Oligodeoxynucleotides on Transforming Growth Factor-1beta Expression and Tumor Necrosis Factor-alpha Content

**FIGURE 8:**
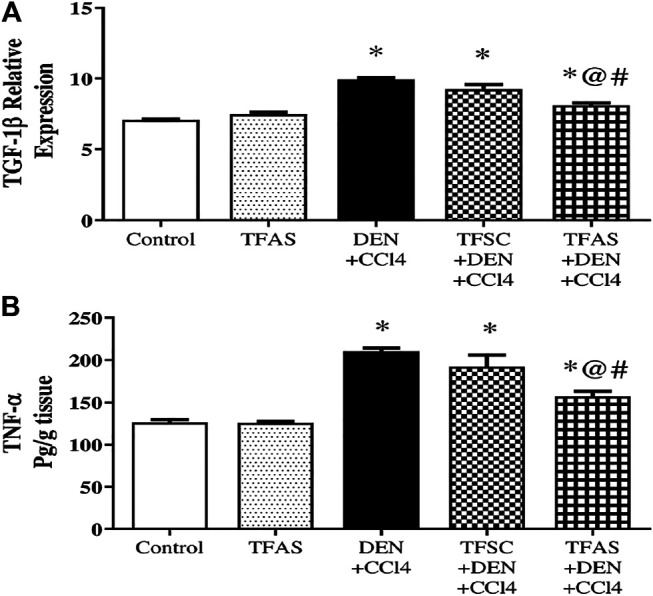
Effect of TF-ODNs on TGF-1β expression and TNF-α content. Data are expressed as mean ± SEM (n = 8). (*), (@) and (#) indicate significant difference from Control, DEN+CCl4 and TFSC+DEN+CCl4, respectively at *P* < 0.05 using one-way ANOVA followed by Tukey-Kramer post-Hoc test. TGF-1β: transforming growth factor beta; TNF-α: tumor necrosis factor alpha; DEN: N-diethyl nitrosamine; CCl4: carbon tetrachloride; TFSC: scrambled tissue factor oligodeoxynucleotides; TFAS: antisense tissue factor oligodeoxynucleotides.

Induction of liver fibrosis by DEN+CCl4 significantly raised liver TGF-1β expression and TNF-α content by 39.44 and 66.64%, respectively compared to the control group. Treatment with TFAS significantly decreased the elevated levels of liver TGF-1β expression and TNF-α content by 18.18 and 25.43%, respectively. Also, TGF-1β expression and TNF-α content significantly decreased by 12.91 and 18.34%, respectively in TFAS treated intoxicated group compared to the TFSC treatment (Figure 8A and Figure 8B).

### Correlation Between Tissue Factor and the Assessed Liver Enzymes Activities, Inflammatory and Fibrotic Markers as Well as Between Protease-Activated Receptors1 and Toll-Like Receptor4 Expression

**FIGURE 9:**
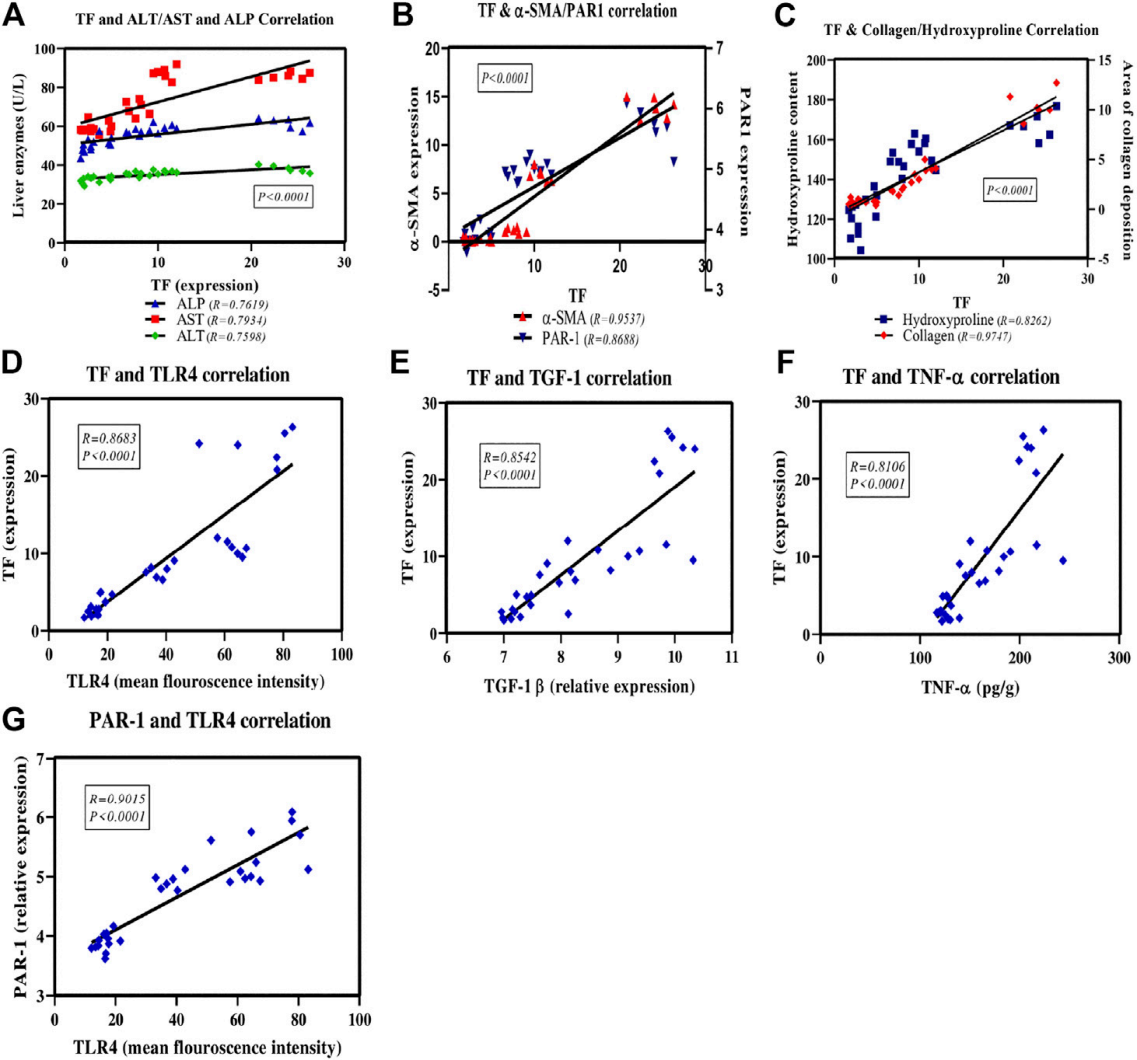
Scatter plots of the correlation between TF and the assessed liver enzymes activities, inflammatory and fibrotic markers as well as between PAR1 and TLR4 expression. The solid lines represent the linear regression and correlation coefficient (r), P is the correlation significance level.

Figure 9 shows the scatter plot of the positive correlation between TF expression and the assessed parameters; ALT, AST and ALP enzyme activities (Figure 9A). There is also a positive correlation between TF expression and the fibrotic markers; α-SMA and PAR1 (Figure 9B) as well as collagen and hydroxyproline (Figure 9C). A positive correlation was displayed also between TF expression and remodeling and inflammatory markers; TLR4 (Figure 9D), TGF-1β (Figure 9E) and TNF-α (Figure 9F). Also, there is a positive correlation between TLR4 and PAR1 expression (Figure 9G) in all study groups.

## Discussion

TF is the transmembrane receptor for FVII/VIIa and the TF-FVIIa complex functions as the primary initiator of coagulation *in vivo*. TF is also recognized as a signaling receptor in different pathological conditions such as angiogenesis, tumor, inflammation and fibrogenesis (Lento et al., 2015). In normal liver, TF has been reported to be expressed primarily by hepatocytes (Sullivan et al., 2013) as well as HSCs, SECs and Kupffer cells (Arai et al., 1995).

In accordance with previous studies, liver fibrosis is induced in rats by co-administration of DEN and CCl4 (Uehara et al., 2013; Uehara et al., 2014; Marrone et al., 2016; Sung et al., 2018). Liver sections excised from animals intoxicated with DEN+CCl4 had intense TF staining in hepatocellular cytoplasm. In accordance with these results, overexpression and a profound role of TF have been reported in different models of chemically induced liver injury (Hammad et al., 2011; Abdel-Bakky et al., 2020), including CCl4-induced fibrosis (Duplantier et al., 2004). Furthermore, Knight et al. (2017) reported that mice with deletion of the cytoplasmic TF domain had amelioration of liver fibrosis compared to wild type mice following 8 weeks of CCl4 exposure. Thus, the overexpression of TF observed in this study and the previously reported implementation of TF in the pathogenesis of different models of liver injuries rose the assumption that targeting TF could be of benefit in managing liver fibrosis.

TF-ODNs were reported to inhibit TF production at both transcriptional and translational levels (Yin et al., 2010; Hammad et al., 2013). In addition, TF-ODNs have been reported to be accumulated predominantly in the rat liver following systemic administration (Nakamura et al., 2002; Shimizu et al., 2014). Indeed, in this study, subcutaneous injection of TFAS significantly inhibited the elevated expression of TF in different liver cells. Similarly, Abdel-Bakky et al. (2011); Abdel-Bakky et al. (2015) and Abdel-Bakky et al. (2020) reported significant changes in TF expression pattern in the liver sections excised from mice intoxicated with thioacetamide and CCl4 upon treatment with TFAS.

In this study animals intoxicated with DEN+CCl4 showed significant elevation in the activities of liver enzymes; ALT, AST and ALP along with severe histopathological deterioration of the liver. These observations are consistent with previous findings that animals treated with CCl4 or DEN+CCl4 showed pronounced biochemical and histopathological fibrotic alterations (Poojari et al., 2010; Uehara et al., 2013; Serag et al., 2019; Tripathy et al., 2020). However, treatment with TFAS significantly improved the abnormal activities of liver enzymes and the histopathological inflammatory and fibrotic changes associated with DEN+CCl4 intoxication. The improvement in enzyme activities is positively correlated with TF expression. In agreement with our findings, blockage of TF expression using TFAS improved histopathological and biochemical deteriorations in chemically intoxicated mice (Hammad et al., 2013; Abdel-Bakky et al., 2015 and Abdel-Bakky et al., 2020). Moreover, Luyendyk et al. (2010) demonstrated that liver macrophage and neutrophil aggregation were significantly reduced in mice with low TF expression compared to heterozygous control mice fed diet deficient in methionine and choline.

The distinguished role of HSCs in liver fibrosis was previously documented (Kisseleva and Brenner, 2021). The extent of HSCs activation and proliferation rates is indicated by the expression of characteristic specific receptors such as α-SMA (Jiang and Torok, 2013; Lin et al., 2018) and excessive production of collagen, where the deposition of collagen fibrils in liver connective tissues provides a hallmark of liver fibrosis development (Hernandez-Gea and Friedman, 2011; Bai et al., 2013). The stability of collagen fibrils is maintained by deposition of hydroxyproline amino acid, which is commonly applied as a marker for fibrogenesis and directly correlated with the total collagen and the stage of liver fibrosis (Ding et al., 2017). In the current study, DEN+CCl4-intoxicated rats showed increased α-SMA expression, collagen production and elevation of liver hydroxyproline content. These results indicated a profound activation of HSCs in response to DEN+CCl4 intoxication. Similarly, previous studies showed that α-SMA expression as well as collagen and hydroxyproline content were significantly elevated in response to injection with CCl4 alone or in combination with DEN (Mansour et al., 2010; Abdel-Moneim et al., 2015; Ahmed and Tag, 2017).

In addition to HSCs, activated Kupffer cells and infiltrated macrophages play a pivotal role in liver fibrogenesis. Activated macrophages release TGF-1β which, among growth factors, is the “master” modulator in fibrogenesis and involved in HSCs activation (Nakazato et al., 2010; Meng et al., 2016). Furthermore, they release, among other proinflammatory cytokines, TNF-α which is one of the early events in many types of liver damage and can activate HSCs and modulate innate immune and inflammatory responses (Gupta et al., 2019). In the present study, liver content of both TGF-1β and TNF-α were significantly increased upon administration of DEN+CCl4, the results that are in accordance with that reported by Tork et al. (2016), Elguindy et al. (2016) and Knight et al. (2017).

The role of coagulation proteases in liver fibrosis was recently explored. One of the primary mechanisms whereby coagulation proteases could contribute to liver fibrosis is through direct activation of HSCs. In support of this hypothesis, we found that the expression of TF is positively correlated with HSCs activation markers; α-SMA, collagen and hydroxyproline. Furthermore, these markers were significantly decreased upon TFAS treatment; these findings are consistent with Knight et al., (2017), as the authors suggested that activation of the cytoplasmic domain of TF promoted liver fibrosis by inducing HSCs activation.

Another mechanism that could underlie the pathological role of TF in liver fibrosis is via stimulation of local inflammatory cell activity. Macrophages were reported to express TF which is upregulated during macrophage maturation and fibrogenesis (Knight et al., 2017). In the present study, TFAS significantly reduced the elevated content of TGF-1β and TNF-α; the main products of inflammatory cells accompanied with fibrosis. Furthermore, our study showed a positive correlation between TF and each of TGF-1β and TNF-α. Consequently, we could assume that in addition to inhibition of HSCs, TFAS decrease TGF-1β and TNF-α production with subsequent inhibition of fibrogenesis and inflammation. In the line with our findings, Knight et al. (2017) reported that deletion of the TF cytoplasmic domain significantly lower gene and protein expression of TGF-1β by activated Kupffer cells.

Since inflammation and coagulation are crosslinked with liver fibrosis and given the principal roles of TLR4 and PAR1 in inflammation and coagulation, respectively, we hypothesized that TLR4-TF-PAR1 axis would be a novel pathway involved in the pathogenesis of liver fibrosis and targeting that pathway could underlie the therapeutic benefits of TFAS.

Several studies have explored the role of PARs in liver fibrogenesis (Borensztajn et al., 2010). In chronic liver disease, HSCs express upregulated PAR1 (Fiorucci et al., 2004). Furthermore, it was found that removal of stellate cell-specific PAR1 produced a 35% reduction in the accumulation of liver collagen (Poole et al., 2020) and PAR1 deficient mice appeared to be protected from CCl4-induced liver fibrosis (Rullier et al., 2008; Kallis et al., 2014). In the line with these findings, the current study showed that PAR1 expression was significantly increased in the DEN+CCl4 intoxicated rats.

TLR4 is a main receptor involved in different inflammatory processes (Lucas and Maes, 2013). A crucial role of TLR4 in fibrogenesis was highlighted in experiment where TLR4 mutant mice showed decreased liver fibrosis in response to toxic agents (Seki et al., 2007; El-Kashef and Serrya, 2019). Furthermore, Liang et al. (2016) demonstrated that HSCs activation and proliferation were prohibited through suppression of TLR4 signaling pathway in DEN-induced liver fibrosis. In accordance with these studies, we reported that liver fibrosis induced by DEN+CCl4 injection was associated with significantly increased expression of TLR4 in the liver tissue.

The contribution of both PAR1 and TLR4 in the beneficial effects of TF blockage was clarified in this study, as rats received TFAS showed a significant reduction in the expression of both PAR1 and TLR4 in liver cells. These findings shed the light on the possibility of crosslinking among the three receptors in controlling liver fibrosis.

Thrombin is produced mainly through cleavage of prothrombin by TF (Mann et al., 2003) and blockage of TF expression resulted in decreased thrombin production (Carraway et al., 2003). Thrombin was reported to produce a dual effect on liver fibrosis via action on PAR1 and through TLR4. Firstly, it was proclaimed that thrombin activates PAR1 on both HSCs and Kupffer cells with subsequent progression of fibrosis (Fiorucci et al., 2004; Rullier et al., 2008). Secondly, thrombin was reported as a critical mediator in LPS-induced liver damage (Rondina et al., 2011) and gut-derived-LPS through TLR4 activation was significantly involved in CCl4-induced liver fibrosis (Seki et al., 2007; Rondina et al., 2011; Wang et al., 2020). Accordingly, blockage of thrombin formation through inhibition of TF could diminish the roles of both PAR1 and TLR4 in the fibrogenic process.

Consequently, based on our findings and the previous studies, we can assume that inhibition of TF expression could censor the downstream production of thrombin with subsequent inhibition of PAR1 and TLR4 mediated fibrogenic and inflammatory process. On the other hand, we can assume that the improvement in the fibrosis observed with TFAS treatment could be reflected in the reduced expression of receptors upregulated under the pathological condition of fibrosis including PAR1 and TLR4.

Notably, the results of this study showed that control oligonucleotides; TFSC resulted in a significant reduction in the expression of TF, PAR1, α-SMA and collagen, although it was significantly less when compared with the effect observed with TFAS. On the other hand, TFSC didn’t affect the level of the other assessed parameters compared to DEN+CCl4 group. These results indicate that TFSC may have some specificity towards the TF mRNA binding site and the decreased expression of PAR1, α-SMA and collagen could be the consequences of TFSC-induced inhibition of TF expression. In support of our explanation, it has been reported that various degrees of downregulation of gene expression have been observed with different types of control oligonucleotides that depend on the nature of the used control oligonucleotides, i.e., its base composition, sequence and/or nature of the backbone (Agrawal, 1996).

## Conclusion

The current study established, for the first time to our knowledge, the potential crosstalk between TF, PAR1 and TLR4 in liver fibrosis. The positive correlation between blockage of TF expression and the downregulation of both PAR1 and TLR4 provides support for the solid crosslink between the receptors. Furthermore, this study reported that blockage of TF expression and gene silencing, using TFAS, reduced liver damage and improved fibrotic changes associated with CCl4+DEN intoxication. These findings offer a platform on which recovery from liver fibrosis could be mediated through targeting TF expression as a key factor in fibrogenesis. Future mechanistic and preclinic studies are recommended to support our findings.

## Data Availability

The original contributions presented in the study are included in the article/Supplementary Material, further inquiries can be directed to the corresponding author.
