# Supplementary figures and images for: Antisense Tissue Factor Oligodeoxynucleotides Protected Diethyl Nitrosamine/Carbon Tetrachloride-Induced Liver Fibrosis Through Toll Like Receptor4-Tissue Factor-Protease Activated Receptor1 Pathway
Source: Front Pharmacol. 2021 May 11;12:676608. doi: 10.3389/fphar.2021.676608 (PMC8144514; doi:10.3389/fphar.2021.676608)

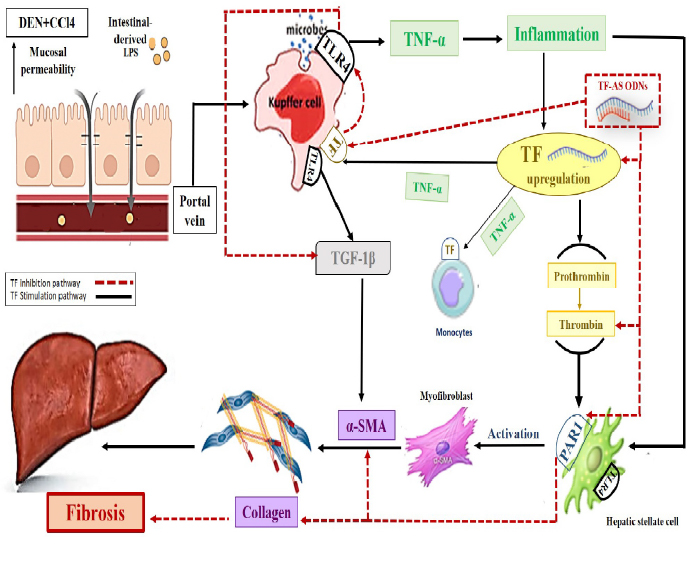

Supplement: Supplementary file 1 [file Image1.JPEG]

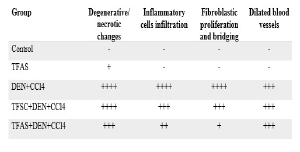

Supplement: Supplementary file 2 [file Image2.JPEG]
